# Mock interviews with video-stimulated recall to prepare medical students for residency interviews

**DOI:** 10.15694/mep.2018.0000168.1

**Published:** 2018-08-14

**Authors:** Lisa Grimaldi, Chengcheng Hu, Paul Kang, Susan Kaib

**Affiliations:** 1 University of Arizona College of Medicine-Phoenix; 2 University of Arizona College of Public Health

**Keywords:** interview, residency match, feedback, video stimulated recall, post-graduate medicine

## Abstract

This article was migrated. The article was marked as recommended.

**Purpose:** The residency interview is the most important factor for residency program directors when deciding on how to rank medical student applicants. With the residency match becoming increasingly competitive, it is more important than ever for students to perform well in this high-stakes interview. Video-stimulated recall (VSR) has been shown to be an effective tool for facilitating reflection on performance and behaviors. As such, we conducted mock interviews with and without video-stimulated recall to gauge its effect on student perceptions of preparedness and confidence for residency interviews.

**Methods:** Students completed a pre-mock interview survey followed by a video recorded interview with faculty. All students received verbal feedback on their performance immediately after the interview. Students were randomized to receive their feedback from their faculty interviewer either while reviewing their video or without the video review. Post-mock interview and post-residency interview surveys were completed. Wilcoxon signed-rank was used to compare median aggregate scores between pre/post surveys. Wilcoxon rank-sum was used to compare pre/post aggregate scores between the video review vs. no-video review groups.

**Results:** 33 of 70 students participated (47%). 14 students (42%) reviewed their video and 19 (58%) received feedback without video. Likert scores for pre- and post-mock interview and post-residency interview surveys revealed median aggregate scores of 10 (interquartile range, or IQR=8,11), 12 (IQR=12,13), and 13 (IQR=12,13) (p <0.001, p<0.001). The change in median aggregate score between pre/post-mock interview surveys in the video review group vs. no-video review group was 3 (IQR=3,5) and 1 (IQR=0,3) (p<0.01) and from pre-mock interview to post-residency interview in the video review vs. no-video review groups was 3 (IQR=3,5) and 2 (IQR=1,4) (p=0.04).

**Conclusions:** The mock interview for residency application improved students’ perceptions of preparedness and confidence. Reviewing the video of the interview while receiving verbal feedback increased students’ confidence in their interview skills.

## Introduction

The residency application process for medical students has become increasingly competitive in recent years. The 2016 Main Residency Match resulted in over 600 US graduating senior medical students lacking residency positions after the Supplemental Offer and Acceptance Program. ( Signer, 2016) There are several reasons for this. Predictions of future physician shortages has led American medical schools to increase class sizes with a goal of an overall increase in enrollment by 30% by 2017. ( AAMC, 2013) In addition, the number of US allopathic medical school seniors participating in the match has grown by 18% between 2005 and 2014. Other groups in the applicant pool including osteopathic graduates and foreign medical graduates have also grown substantially in recent years. ( NRMP, 2014)

Despite the considerable increase in the number of medical school graduates over the past 10 years, the number of residency positions has remained relatively constant. As a result, in the 2016 residency match there were 42,370 registered applicants competing for only 30,750 available positions making the 2016 match the most competitive to date. ( NRMP, 2016) It is therefore incumbent upon medical schools to prepare senior students as much as possible to maximize their chances of success in the match.

The interview has been shown to be possibly the most important factor in residency program directors’ decision making and a significant influence on the ranking order of residency candidates. ( Gong *et al.*, 1984) Interactions with faculty and house staff during the interview were cited as two of the most important factors in ranking a candidate according to the 2014 National Residency Matching Program Survey of Program Directors. ( NRMP, 2014) These data suggest that improving interviewing skills may favorably affect candidates’ chances of securing a residency position.

Despite this, there is little in the literature to determine the most effective way to prepare students for residency interviews. Ryan *et al.* demonstrated that conducting mock interviews for senior medical students in a group setting could improve students’ perceptions of preparation and confidence for residency interviews. ( Ryan, Ewing and O’Brien, 2014)Several studies in the pharmacy literature have shown that implementation of a mock interview program including individual practice interviews with faculty and verbal feedback can not only help students feel more confident and prepared for the real interview process( Caballero *et al.*, 2012), but can potentially improve match success rates. ( Koenigsfeld *et al*., 2012) But despite the high-stakes nature of the residency interview, to the authors’ knowledge, there is no standardized approach to preparing students and practices vary widely across medical schools.

**Figure 2.**
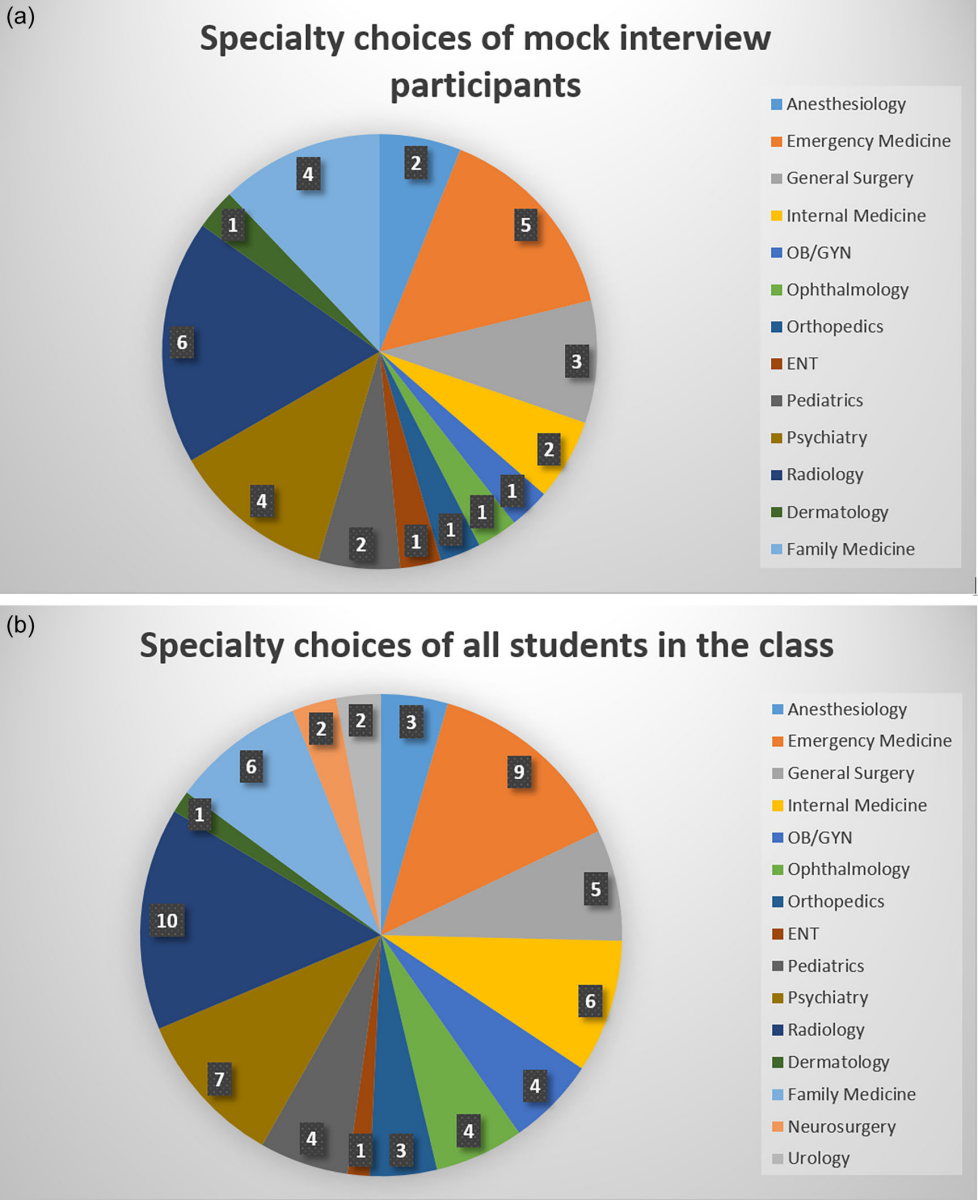
a). Specialty choices of students participating in the mock interviews; Numbers indicate how many students interviewed for each specialty. b). Specialty choices of all students in the class; Numbers indicate how many students planned to apply to each specialty.

As noted above, limited evidence does exist that practice interviews are of value in preparing medical students for residency interviews. However, it is unclear what is the optimal way to provide feedback and how best to encourage reflection on interview performance. Previous studies in medical education have shown that giving immediate verbal feedback is a valuable learning tool and can improve preparedness, confidence and performance in various clinical and educational settings. ( Ende, 1983) ( Hodder *et al.*, 1989) ( Garner *,* Gusberg and Kim, 2014) Video stimulated recall (VSR), or the review of video-recorded events of an individual’s behavior has been used extensively in social science research and has been shown to be helpful in facilitating reflection on social behaviors. ( Lyle, 2003) In educational research, VSR has been shown to be an effective tool for enabling instructors and students to articulate their thinking and feelings and even uncover cognitive processes. ( Powell, 2005) Furthermore, evidence from the psychology literature suggests that individuals can learn more effectively by watching their own performances. ( Knoblich and Sebanz, 2006) However, to the authors’ knowledge, no evidence exists demonstrating the effectiveness of VSR in the preparation of medical students for residency interviews.

In our study, we established a voluntary mock interview session for senior medical students at our institution. We then utilized VSR in providing feedback to a subset of the students. Our aims were to: (1) determine whether conducting individual mock residency interviews for senior medical students increases perceived preparedness, confidence, and performance in residency interviews; and (2) to determine if receiving verbal feedback while reviewing the video recording of the mock interview with faculty immediately after the mock interview enhances the effectiveness of the mock interview experience.

## Methods

**Study design:** The study was approved by and conducted in accordance with the University of Arizona Institutional Review Board - Human Subjects Protection Program. All senior medical students who were on target to graduate and planned to enter the 2016 residency match were invited by email to participate in a mock interview activity prior to the start of the residency interview season. Students received a disclosure attached to the email stating that participation in the investigation was voluntary and they had the option of participating in the mock interview activity without participating in the research study. Students who agreed to the disclosure statement were directed to the pre-mock interview survey (Survey #1) assessing demographic information, their intended residency specialty choice, and their perceptions about their preparedness and confidence level for residency interviews using a 5-point Likert scale ( Appendix A). After completing the survey, they were directed to a web-based sign-up for the mock interview activity. Students who did not agree to the disclosure bypassed the survey and were directed to a web-based sign up for the mock interview. Students completing the survey were assigned a unique identifier which was used on all surveys going forward so that all student information was de-identified.

The mock interview activity was conducted on two separate evenings to maximize students’ availability and students could choose which night to attend. Each mock interview activity consisted of two components: a mock interview portion and a roundtable discussion with faculty and local residency program directors so that students could ask specialty-specific questions about the residency interview process. For the mock interview, each student had a 20-minute, individual mock interview with a faculty member who may or may not have been from the students’ chosen specialty. Students were encouraged to bring their CV and personal statement to the mock interview. Each interview was followed immediately by verbal feedback with the faculty interviewer. All interviews were video-recorded. Students were alternately assigned in a randomized fashion to either view their video with their faculty interviewer immediately after the mock interview while receiving feedback (video review group), or to receive verbal feedback without reviewing their video (no-video review group). All faculty members were given a standardized feedback form ( Appendix E) and were instructed to give feedback to the students addressing all of the elements indicated on the form. Since watching the video would require additional time, the video review group was given 20 minutes for feedback in order to allow time to cover all of the feedback elements and the no-video review group was given 10 minutes for feedback.

Immediately following feedback, students were asked to complete a post-mock interview survey (Survey #2) again assessing perceptions about confidence and preparedness for residency interviews and to gauge the effectiveness of the feedback they received ( Appendix B).

A third survey was administered after the completion of the residency interview season (Survey #3) to assess students’ perceptions of preparedness, confidence, and performance in their actual residency interviews ( Appendix C). All three surveys contained the same three questions to assess students’ perceptions of preparedness, knowing what types of questions to expect, and confidence for residency interviews using a 5-point Likert scale (1=Strongly disagree, 5=Strongly agree).

All participating students and faculty received the following information prior to the mock interview session: 1) details about the format of the session 2) a list of commonly asked interview questions ( Appendix D), and 3) a PowerPoint presentation providing tips for successful interviewing and links to interviewing skills resources.

The faculty who were invited to participate in the event were from the departments in which students planned to pursue residencies, as well as members of the Student Affairs and Admissions teams. The individuals chosen as faculty interviewers all had experience both with interviewing student or residency candidates and with giving feedback to students and house staff. Faculty who were assigned to interview students in the video review group all had experience in providing feedback to students using video review as instructors in the Doctoring course for first and second year medical students where standardized patients and video review are used to teach medical students history taking and physical exam skills. Since the number of faculty who were deemed qualified to do this were limited, the number of students who were randomized to the video review group had to be limited accordingly.

**Statistical methods:** For students who participated in the mock interview program, student characteristics including age, gender, and specialty were summarized using median and interquartile range (IQR) for continuous variables and frequency and proportion for categorical variables, and were compared between the subjects who received immediate video-based faculty review and those who did not, using either the Wilcoxon rank-sum test (for continuous variables like age) or the Fisher’s exact test (for categorical variables like gender and specialty).

All three surveys shared three identical questions assessing preparedness, knowing what type of questions to expect, and confidence in residency interviews (Questions 4, 5 and 6 of Survey #1, Questions 3, 4 and 5 of Survey #2, and Questions 6, 7 and 8 of Survey #3). For each survey an aggregate score was calculated as the sum of the responses to these three questions, with the five levels of the response for each question coded as integers 1 through 5. To evaluate the effect of the mock interview, for subjects who completed all three surveys, the Wilcoxon signed-rank test was used to compare the baseline pre-mock interview aggregate score with the post-mock interview score and also separately with the post-residency interview score. The tests were also performed separately for the score of each individual question. To evaluate the effect of the immediate video review, the Wilcoxon rank-sum test was used to compare the change in aggregate score from baseline to the post-mock interview survey between the two groups of subjects receiving or not receiving the video review. All statistical tests were two-sided with significance level 0.05.

## Results/Analysis

**Figure 1.**
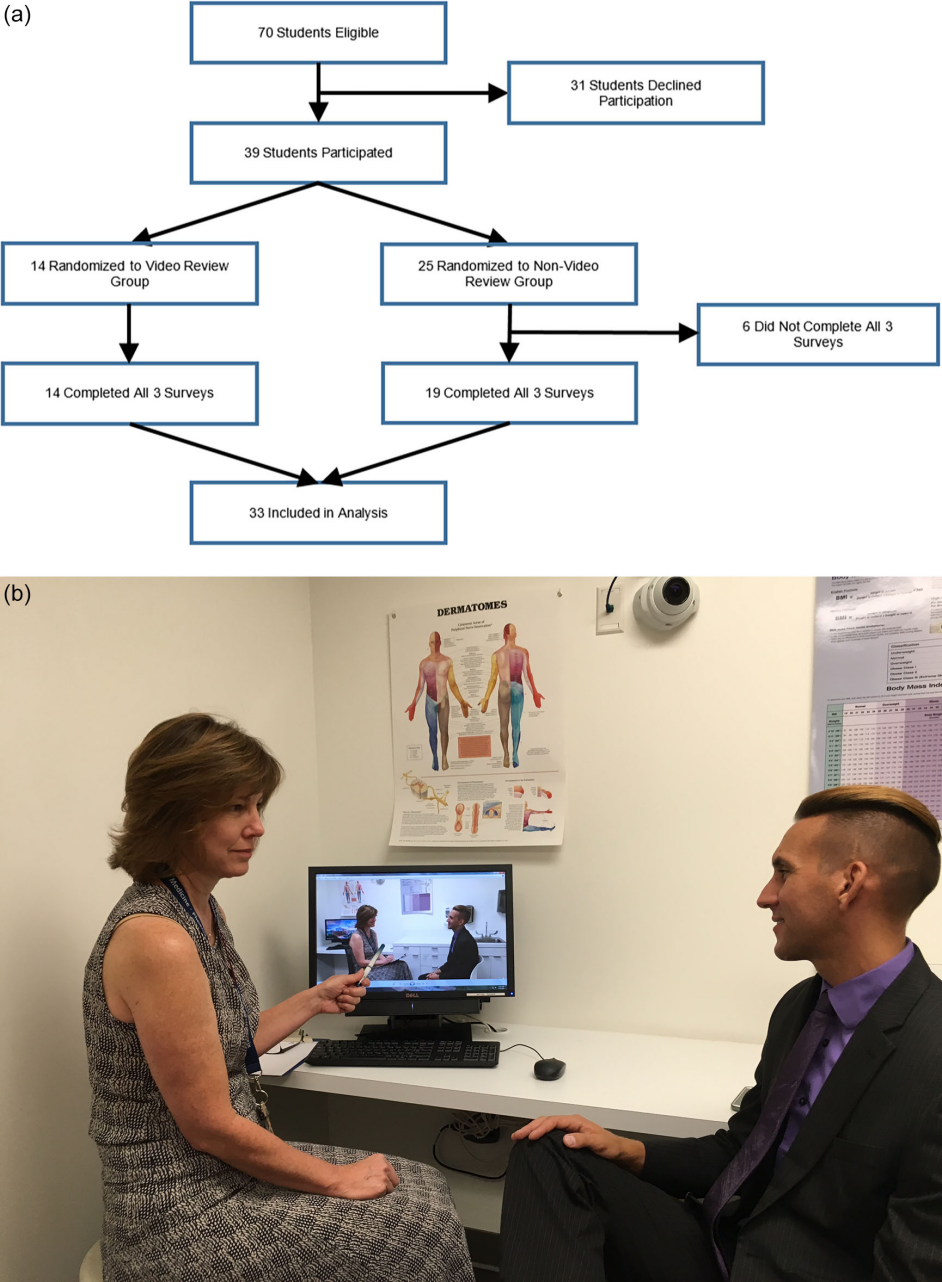
a). Student participation and randomization. b). A student receiving verbal feedback with video review (both individuals gave consent for this image to be published)

Twenty-eight faculty members from the departments of Obstetrics and Gynecology, Internal Medicine, Family and Community Medicine, Child Health, Anesthesiology, Radiology, Psychiatry, Emergency Medicine, Radiation Oncology, and Surgery as well as members of the Student Affairs and Admissions teams participated in one or both of the mock interview sessions by conducting interviews, being a part of the roundtable discussion or both. A total of 39 of the 70 eligible students (56%) participated in the mock interview activity and 33 students (85%) completed all 3 surveys. Among these 33, 14 students (42%) were randomized to the video review group and 19 students (58%) were randomized to the no-video review group ( Figure 1).

Complete demographic information regarding the participants can be found in Table 1. The median age of students was 27 (IQR=26, 28) and 16 students (49%) were men and 17 (51%) were women. Thirty out of the 33 participants (91%) had done no preparation prior to the mock interview. Students who participated planned to apply for residencies in the following specialties: anesthesiology, emergency medicine, general surgery, internal medicine, obstetrics and gynecology, ophthalmology, orthopedic surgery, otolaryngology, pediatrics, psychiatry, radiology, dermatology, and family medicine ( Figure 2). There were no significant differences in baseline characteristics between the students in the video review and the no-video review groups.

**Table 1.** Demographic information and baseline characteristics

Characteristics	Overall N=33	No Video Review N=19	Video Review N=14	P-Value ^1^
Age in years, median (IQR)	27 (26, 28)	27 (25, 31)	26 (26, 27)	0.18
Gender (N, %) Male Female	16 (48.5) 17 (51.5)	10 (52.6) 9 (47.4)	7 (50.0) 7 (50.0)	1.00
Have you been preparing for residency interviews using other resources (N, %) Yes No	3 (9.1) 30 (90.9)	3 (15.8) 16 (84.2)	0 (0) 14 (100)	0.24
Specialty Interviewed For (N, %) Anesthesiology Emergency Medicine General Surgery Internal Medicine OB/Gyn Ophthalmology Orthopedic Otolaryngology Pediatrics Psychiatry Radiology Dermatology Family Medicine	2 (6.1) 5 (15.2) 3 (9.1) 2 (6.1) 1 (3.0) 1 (3.0) 1 (3.0) 1 (3.0) 2 (6.1) 4 (9.1) 6 (21.2) 1 (3.0) 4 (12.1)	2 (10.5) 4 (21.1) 1 (5.3) 0 (0) 1 (5.3) 1 (5.3) 1 (5.3) 1 (5.3) 1 (5.3) 1 (5.3) 4 (21.1) 1 (5.3) 1 (5.3)	0 (0) 1 (7.1) 2 (14.3) 2 (14.3) 0 (0) 0 (0) 0 (0) 0 (0) 1 (7.1) 3 (21.4) 2 (14.3) 0 (0) 3 (31.4)	0.41

^1^ Wilcoxon rank-sum comparing continuous variables and Fisher’s Exact comparing categorical variables.

**Figure 3.**
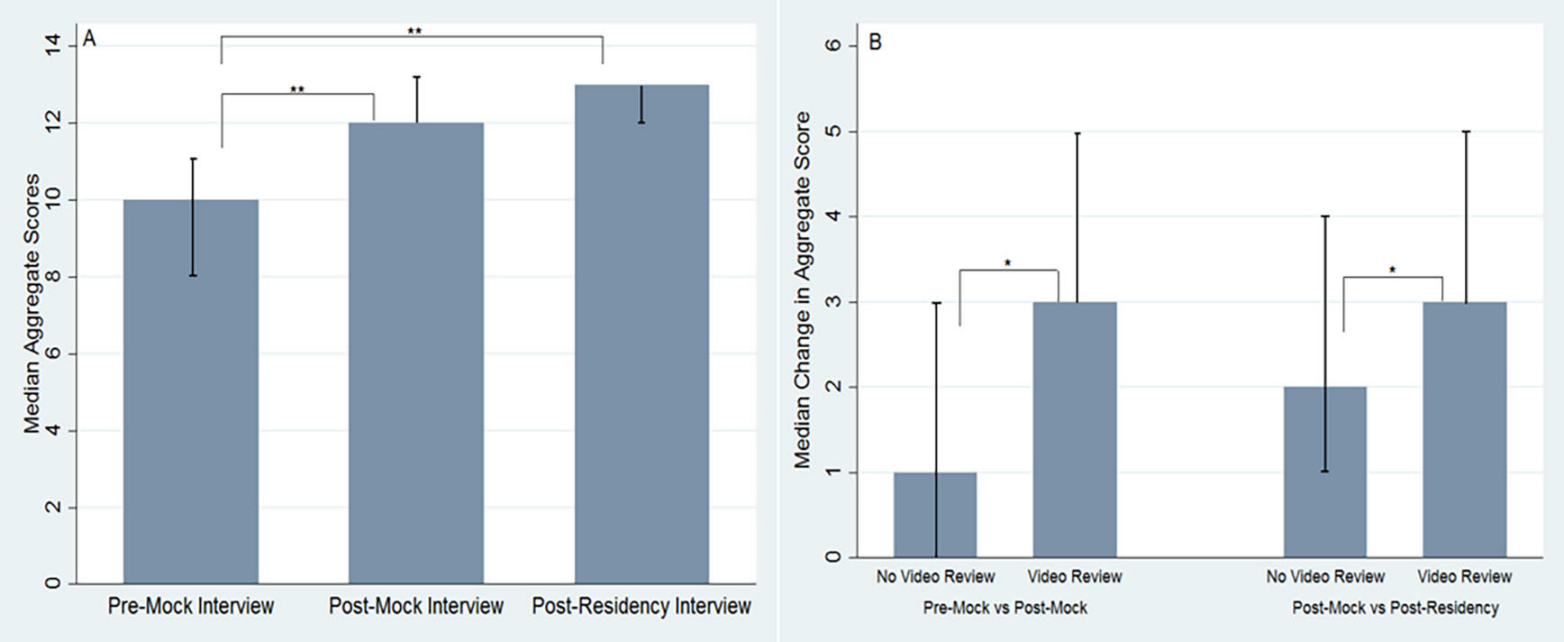
a) Median (IQR) aggregate Likert scores for Pre-Mock Interview, Post-Mock Interview, and Post-Residency Interview surveys respectively. b) Change in aggregate score from Pre-Mock Interview to Post-Mock Interview and Post-Residency Interview surveys respectively.

Our primary analysis focused on the three questions common to all three surveys: assessing preparedness, knowing what type of questions to expect, and confidence in residency interviews. The Likert scores for each question were compared from pre-mock interview, post-mock interview, and post-residency interview surveys as were the total aggregate Likert scores for those three questions from pre-mock interview, post-mock interview, and post-residency interview surveys ( Table 2).

**Table 2.** Comparison of preparedness, question expectation, confidence, and the aggregate score at different time points

Survey Question	Pre-Mock Interview	Post-Mock interview	Post-Residency interview	P-Value ^1^	P-Value ^2^
	N=33	N=33	N=33		
	N (%)	N (%)	N (%)		
I feel prepared for residency interviews. Strongly Disagree Disagree Neutral Agree Strongly Agree	0 (0) 9 (27.3) 11 (33.3) 13 (39.4) 0 (0)	0 (0) 1 (3.0) 5 (15.1) 22 (66.7) 5 (15.2)	0 (0) 0 (0) 0 (0) 26 (60.6) 13 (39.4)	<0.001	<0.001
I know what type of questions to expect at residency interviews. Strongly Disagree Disagree Neutral Agree Strongly Agree	0 (0) 13 (39.4) 9 (27.3) 11 (33.3) 0 (0)	0 (0) 0 (0) 3 (9.1) 24 (72.7) 6 (18.2)	0 (0) 0 (0) 1 (3.0) 26 (78.8) 6 (18.2)	<0.001	<0.001
I feel confident about my ability to perform well in residency interviews. Strongly Disagree Disagree Neutral Agree Strongly Agree	0 (0) 2 (6.1) 10 (30.3) 19 (57.5) 2 (6.1)	0 (0) 0 (0) 3 (9.1) 17 (51.5) 13 (39.4)	0 (0) 0 (0) 1 (3.0) 21 (63.4) 1 (33.3)	<0.001	<0.001
Aggregate Score, median (IQR)	10 (8, 11)	12 (12, 13)	13 (12, 13)	<0.001	<0.001

^1^ Wilcoxon signed-rank test comparing Post-Mock Interview scores vs Pre-Mock Interview scores.

^2^ Wilcoxon signed-rank test comparing Post-Residency Interview scores vs Pre-Mock Interview scores

Median total aggregate Likert scores for the pre-mock interview and post-mock interview surveys were 10 (IQR=8, 11) and 12 (IQR=12, 13) respectively (p<0.001). Median total aggregate Likert scores for the post-residency interview survey was 13 (IQR=12, 13) (p<0.001 compared to pre-mock interview) ( Figure 3a). The change in the median aggregate Likert score from the pre-mock interview to post-mock interview surveys in the video review group vs. the no-video review group was 3 (IQR=3,5) and 1 (IQR=0,3) respectively (p<0.01). The change in the median aggregate Likert scores from the pre-mock interview to post-residency interview surveys in the video review group vs. the no-video review group was 3 (IQR=3,5) and 2 (IQR=1,4) respectively (p=0.04) ( Table 3, Figure 3b).

**Table 3.** Comparison of change in preparedness, question expectation, confidence, and the aggregate score by video review status

Characteristics	Video Review	No Video Review	P-Value ^1^
	N=14	N=19	
	N (%)	N (%)	
I feel prepared for residency interviews.			0.86
Strongly Disagree Disagree Neutral Agree Strongly Agree	0 (0) 1 (7.14) 2 (14.3) 8 (57.1) 3 (21.4)	0 (0) 0 (0) 3 (15.8) 14 (73.7) 2 (10.5)	
I know what type of questions to expect at residency interviews			0.57
Strongly Disagree Disagree Neutral Agree Strongly Agree	0 (0) 0 (0) 2 (14.3) 8 (57.1) 4 (28.6)	0 (0) 0 (0) 1 (5.3) 16 (84.2) 2 (10.5)	
I feel confident about my ability to perform well in residency interviews.			0.68
Strongly Disagree Disagree Neutral Agree Strongly Agree	0 (0) 0 (0) 1 (7.1) 7 (50.0) 6 (42.9)	0 (0) 0 (0) 2 (10.5) 10 (52.6) 7 (36.8)	
Change in Pre/Post Mock Interview aggregate scores, median (IQR) Change in Pre/Post Residency Interview aggregate scores, median (IQR)	3.0 (3, 5) 3.0 (3, 5)	1.0 (0, 3) 2.0 (1, 4)	<0.01 0.04

^1^ Wilcoxon rank-sum comparing median change in aggregate score between video review and no video review groups.

Additional exploration into the responses to the questions on the post-mock interview survey showed a trend for students in the video review group to have a more positive assessment of the effectiveness of the mock interview activity and the feedback they received. Students in the video review group were more likely to feel that their interviewing skills improved as a result of the mock interview session (57% strongly agreeing in the video review group vs 42% in the no video review group) and were more likely to feel that the feedback they received was helpful (93% strongly agreeing in the video review group vs 79% in the no video review group). These values did not reach statistical significance (p =0.71, p=0.28)( Table 4).

**Table 4.** Comparison of response to additional post-mock interview survey questions by video review status

Characteristics	Total Student Population (n=33)	Video Review N=14	No Video Review N=19	P-Value ^1^
	N (%)	N (%)	N (%)	
How much time did you prepare for this mock interview? No Preparation 1-2 Hours 3-4 Hours >4 Hours	18 (54.5) 14 (42.4) 1 (3.1) 0 (0)	10 (71.4) 4 (28.6) 0 (0) 0 (0)	8 (42.1) 10 (52.6) 1 (5.3) 0 (0)	0.08
I performed well in this mock interview.				0.42
Strongly Disagree Disagree Neutral Agree Strongly Agree	0 (0) 1 (3.1) 4 (12.1) 20 (60.6) 8 (24.2)	0 (0) 1 (7.1) 1 (7.1) 10 (71.4) 2 (14.4)	0 (0) 0 (0) 3 (15.8) 10 (52.6) 6 (31.6)	
I feel my interviewing skills have improved because of this mock interview session. Strongly Disagree Disagree Neutral Agree Strongly Agree	0 (0) 0 (0) 2 (6.1) 15 (45.5) 16 (48.5)	0 (0) 0 (0) 2 (14.3) 4 (28.6) 8 (57.1)	0 (0) 0 (0) 0 (0) 11 (57.9) 8 (42.1)	0.71
The feedback I received from the faculty member following the mock interview was helpful. Strongly Disagree Disagree Neutral Agree Strongly Agree	0 (0) 0 (0) 0 (0) 5 (15.2) 28 (84.8)	0 (0) 0 (0) 0 (0) 1 (7.1) 13 (92.9)	0 (0) 0 (0) 0 (0) 4 (21.1) 15 (78.9)	0.28
I have identified specific areas where I can improve my interviewing skills prior to my residency interviews. Strongly Disagree Disagree Neutral Agree Strongly Agree	0 (0) 1 (3.0) 1 (3.0) 11 (33.3) 20 (60.7)	0 (0) 1 (7.1) 0 (0) 4 (28.6) 9 (64.3)	0 (0) 0 (0) 1 (5.3) 7 (36.8) 11 (57.9)	0.78

^1^ Wilcoxon rank-sum comparing secondary outcomes between video review and no video review groups.

## Discussion

The residency interview is a high-stakes evaluation as it represents a key determinant for residency program directors to select candidates in an increasingly competitive match environment. Several recent studies have investigated the use of a mock interview program to prepare students but with some notable differences from our study in both design and outcomes. In 2014, Ryan *et al.* conducted practice interviews for senior medical students in groups of four with one student undergoing the interview while the other three students in the group observed. They concluded that conducting mock interviews in a group setting was an effective and time-efficient way to improve students’ perceptions of confidence and preparedness for residency interviews but they did not investigate if there was any longer term benefit of the activity with follow up surveys. In 2016, a similar mock interview program was implemented but with individual mock interviews. They found that such a program was feasible and may have improved students’ comfort and confidence level in the residency interview process. ( Multerer *et al.*, 2016) However, the program was offered only to students interested in pediatrics and therefore was not representative of the medical school class as a whole. Another similar mock interview program was conducted at the Medical College of Wisconsin in 2016 and demonstrated that students felt that individual mock interviews followed by verbal feedback helped them identify their strengths and weaknesses and that the students’ perceptions of the benefit of the program were still present five months later. They further noted that the group of students who participated in the mock interview program demonstrated a higher match rate than those who did not, suggesting a potential benefit not only in perceptions but in actual interview performance. ( Hueston and Holloway, 2016) The validity of this conclusion is unclear however since the mock interview program was voluntary and students who participated were self-selected therefore creating a potential volunteer bias that could have affected these results.

To the authors’ knowledge, ours is the first study to evaluate the use of immediate video review with verbal feedback from faculty after an individual mock interview to prepare students for residency interviews. Our data demonstrate that conducting individual mock interviews with immediate verbal feedback is an effective approach for improving students’ perceptions of confidence and preparedness for residency interviews, consistent with what previous studies have shown. Our data further suggest that the addition of video review to the feedback process, although challenging because of the need for more time and resources, may be very worthwhile since the students who received feedback on their mock interview performance with video review demonstrated greater perceptions of confidence and preparedness for residency interviews both immediately after the mock interview activity and after they completed their actual residency interviews. Furthermore, students who received feedback with video review trended toward being more likely to feel that their interviewing skills had improved and that the feedback they received was helpful. These data suggest that the process of watching oneself while receiving feedback may have a greater potential to effect change in performance and behavior.

These findings are consistent with what has long been suspected. The importance of feedback towards the acquisition of clinical skills has been clearly demonstrated in the literature and these same principles likely apply to the acquisition of interviewing skills as well. In *JAMA* in 1983, Ende commented that “feedback occurs when a student is offered insight into what he or she actually did as well as the consequences of his or her actions. This insight is valuable insofar as it highlights the dissonance between the intended result and the actual result, thereby providing impetus for change.” He further stated that “as a compendium of cognitive, psychomotor, and affectual behaviors, clinical skill is easier demonstrated than described” and that “like ballet, it is best learned in front of a mirror.”

The neurobiology behind the use of video review to promote self-reflection and change in behavior has also been investigated in the psychology literature. For example, Knoblich and Sebanz showed that dancers demonstrate increased brain activity when watching videos of themselves dancing rather than watching others perform the same dance. ( Knoblich and Sebanz, 2006) This suggests that there is selective activation of the internal repertoire that allows us to distinguish our own actions from those of others and that seeing ourselves in action has a greater resonance in our neurons.

This concept has been similarly demonstrated in the literature across various disciplines where VSR has been shown to be an effective educational tool. For example, in 2015 Chen *et al.* used VSR to help attending surgeons reflect on the guidance they were giving to surgical residents in the operating room (OR), which is a key element in developing resident autonomy and directly influences resident confidence and the ability to operate independently when transitioning from resident to attending surgeons. Attending surgeons tended to underestimate the amount of guidance they were providing to residents in the OR and the residents had a significantly different perception about the amount of OR guidance they were receiving. VSR facilitated attending surgeons’ reflection on their behavior and decision making in the OR that influenced the amount of guidance they were providing to their trainees. ( Chen, Williams and Smink, 2015) VSR has also been used in the primary care realm, where it has been implemented to help primary care physicians reflect on their consultations with patients to identify barriers to communication that they did not realize existed and foster stronger doctor-patient relationships. ( Paskins, McHugh and Hassell, 2014)

In keeping with these studies, our data suggest that receiving verbal feedback while simultaneously reviewing the video recording of their mock interview aided the students’ reflection on their verbal and non-verbal communication leading to a greater understanding of their behaviors and social interaction in the mock interview. This likely resulted in what was perceived by the students to be more meaningful feedback and identification of specific areas for improvement in interviewing skills leading to a greater sense of confidence and preparedness for their residency interviews.

Several limitations of our study must be acknowledged. Since the mock interview activity consisted of both the roundtable discussion with residency program directors and the individual mock interviews with feedback, it is difficult to know if the students’ increased perceptions of confidence and preparedness were from the mock interview itself or from the activity as a whole. Also, our sample size was small and did not allow for detailed regression to adjust for potential confounders that could explain the differences observed between the video review and no-video review groups. However, there was no evidence to suggest imbalance in the randomization as shown in Table 1. One potential confounder is the fact that the students randomized to the video review group all interviewed with and received feedback from faculty who were experienced in providing feedback using video review. Therefore, the enhanced effectiveness of the experience for the video review group could be due to the increased skill level and experience in giving feedback that these faculty had, rather than the video review itself. Most notably, our study was designed only to assess student perceptions of their confidence and preparedness - not their actual performance in residency interviews or match success. Since the mock interview program was voluntary, there is also a potential volunteer bias. Despite these limitations, we feel that our study is the first to demonstrate the feasibility and utility of VSR in the process of preparing medical students for residency interviews.

## Conclusion

In light of the highly competitive residency match climate, attempts to best prepare senior medical students for success in the match will remain of the utmost importance in the coming years. Our study reveals that a mock interview program including individual mock interviews and verbal feedback with faculty is a feasible and effective approach to improve students’ confidence and perceived preparedness for residency interviews. The increased resource utilization required to provide feedback with video review seems to be justified given the enhanced effectiveness of the mock interview activity that it provided. Future studies designed to assess interview performance and outcomes by match success rate would be most helpful.

## Take Home Messages

• Students must perform well in residency interviews for success in the match.
• There is no standardized approach to prepare students.
• Mock interviews followed by verbal feedback increased students’ perceptions of confidence and preparedness.
• Video review increased students’ confidence in their interviewing skills.
• Mock interviews with video review may be an effective way to prepare medical students for residency interviews.

## Notes On Contributors

LISA M. GRIMALDI, MD is a Pediatric Intensive Care physician at Phoenix Children’s Hospital and Director of the Pulmonary/Renal/Acid-Base Block at the University of Arizona College of Medicine-Phoenix. She was previously a Career and Professional Advisor. Dr. Grimaldi is an Associate Professor in the Department of Child Health.

CHENGCHENG HU, PhD, is Associate Professor, Public Health and Director, Biostatistics, Phoenix Campus, at the UA Mel and Enid Zuckerman College of Public Health. He directs the Biostatics and Study Design Service at the UA College of Medicine-Phoenix, and is involved with research in a wide range of medical areas.

PAUL KANG, MPH is a biostatistician for the University of Arizona College of Medicine-Phoenix and College of Public Health- Phoenix. His main responsibility is to provide statistical support to both students and faculty members. Paul has been involved with several publications with many more manuscripts still in progress.

SUSAN KAIB, MD is the Associate Dean of Student Affairs at the University of Arizona College of Medicine - Phoenix. Previously, she was a Career and Professional Advisor in the Department of Student Affairs, Asst. Director of Doctoring, and holds the title of Associate Professor of Family, Community and Preventive Medicine.

## Appendices

### Appendix A. Survey #1 - Pre-Mock Interview Survey

What are the first 3 letters of your mother’s first name? __________

What are the last 2 digits of your phone number? __________

1. What is your gender?

Male Female

2. What is your age? __________
3. What specialty are you planning on interviewing for? __________
4. I feel prepared for residency interviews.

Strongly disagree Disagree Neutral Agree Strongly agree

5. I know what type of questions to expect at residency interviews.

Strongly disagree Disagree Neutral Agree Strongly agree

6. I feel confident about my ability to perform well in residency interviews.

Strongly disagree Disagree Neutral Agree Strongly agree

7. Have you been preparing for residency interviews using other resources?

Yes No

8. If you have been preparing for residency interviews with other resources, what resources have you been using? Check all that apply.

Careers in Medicine online resources

Other online resources - please list ____________

Practice interviews with friends or faculty members

Other - please list _____________

### Appendix B. Survey #2 - Post-Mock Interview Survey

What are the first 3 letters of your mother’s first name? __________

What are the last 2 digits of your phone number? __________

1. How much time did you prepare for this mock interview?
  No preparation 1-2 hours 3-4 hours >4 hours
2. I performed well in this mock interview.
  Strongly disagree Disagree Neutral Agree Strongly agree
3. I feel prepared for residency interviews.
  Strongly disagree Disagree Neutral Agree Strongly agree
4. I know what type of questions to expect at residency interviews.
  Strongly disagree Disagree Neutral Agree Strongly agree
5. I feel confident about my ability to perform well in residency interviews.
  Strongly disagree Disagree Neutral Agree Strongly agree
6. I feel like my interviewing skills have improved because of this mock interview session.
  Strongly disagree Disagree Neutral Agree Strongly agree
7. The feedback I received from the faculty member following the mock interview was helpful.
  Strongly disagree Disagree Neutral Agree Strongly agree
8. I have identified specific areas where I can improve my interviewing skills prior to my residency interviews.
  Strongly disagree Disagree Neutral Agree Strongly agree
9. Did you review your video with faculty immediately after your mock interview and receive feedback?
  Yes No

### Appendix C. Survey #3 - Post-Residency Interview Survey

What are the first 3 letters of your mother’s first name? __________

What are the last 2 digits of your phone number? __________

1. What is your gender?
  Male Female
2. What is your age? _________
3. What specialty did you interview for? _____________
4. On average, how much time did you spend preparing for each residency interview?
  No preparation 1-2 hours 3-4 hours >4 hours
5. I believe I performed well in my residency interviews.
  Strongly disagree Disagree Neutral Agree Strongly agree
6. I felt prepared for my residency interviews.
  Strongly disagree Disagree Neutral Agree Strongly agree
7. The questions I was asked in my residency interviews were what I was expecting.
  Strongly disagree Disagree Neutral Agree Strongly agree
8. I felt confident in my residency interviews.
  Strongly disagree Disagree Neutral Agree Strongly agree
9. Did you participate in a Student Affairs mock interview session prior to residency interviews?
  Yes No (If Yes please complete questions # 10-13)
10. Did you review your mock interview video with a faculty member immediately after the interview and receive feedback?
  Yes No
11. Did you review your mock interview video on your own at a later time?
  Yes No
12. The feedback I received from the faculty member following the mock interview was helpful in preparing me for residency interviews.
  Strongly disagree Disagree Neutral Agree Strongly agree
13. The questions I was asked in the mock interview prepared me for residency interviews.
  Strongly disagree Disagree Neutral Agree Strongly agree

### Appendix D. Frequently asked interview questions: From AAMC Careers in Medicine ^R^

1. Tell me about yourself.
2. Why did you become a doctor?
3. How would your friends describe you?
4. What are your strengths and weaknesses?
5. Why are you interested in our program?
6. What are you looking for in a program?
7. Why should we choose you?
8. Can you tell me about this deficiency on your record?
9. Why are you interested in this specialty?
10. Tell us about your research experience.
11. If you could not be a physician, what career would you choose?
12. What do you see yourself doing in the future?
13. What leadership roles have you held?
14. What do you do in your spare time?
15. What was your favorite course in medical school?
16. Why did you choose this specialty?
17. What are your goals?
18. Are you interested in academic or in clinical medicine?
19. Do you want to do research?
20. What was the most interesting case that you have been involved in?
21. Do you plan to do a fellowship?
22. What is your most important accomplishment?
23. What motivates you?
24. What will be the toughest aspect of this specialty for you?
25. If you could do medical school over again, what would you change?
26. What do you think you can contribute to this program?
27. Do you see any problems managing a professional and a personal life?
28. Are you prepared for the rigors of residency?
29. How much did lifestyle considerations fit into your choice of specialty?
30. Describe the best/worst attending with whom you have ever worked.
31. What is the greatest sacrifice you have already made to get to where you are?
32. What problems will our specialty face in the next 5-10 years?
33. How would you describe yourself?
34. List three abilities you have that will make you valuable as a resident in this specialty.
35. Describe a particularly satisfying or meaningful experience during your medical training. Why was it meaningful?
36. What is one event you are proudest of in your life?
37. What was the most difficult situation you encountered in medical school?
38. What clinical experiences have you had in this specialty?
39. How well do you take criticism?
40. What questions do you have for me?

### Appenxix E. Faculty to Student Feedback: Mock Interviews 2015-2016

Class of 2016

Faculty Member: _________________________________ Circle date: Sept 24, 2015 Oct. 6, 2015

Student Name: __________________________________

**Feedback Flow *:*** Faculty asks student what went well and what they’d like to redo given the chance (or what didn’t go well). Then move to the following areas to review during feedback.

1. Communication, non-verbal
  a. Proper use of eye contact, smiling, nodding when appropriate, etc.
  b. Discuss any observed blocking behaviors such as, arms crossed, leaning away from interviewer, lack of eye contact, nervous habits, distracting behaviors, etc.
  c. Comes across genuine in responses
2. Communication, verbal
  a. Proper use of English grammar, complete answers without repetition or fragmentation of thoughts
  b. Discuss use of filler words or slang (um, like, cool, yay, etc.)
3. Content
  a. Discuss if responses to questions were well thought-out and clear
  b. Discuss if the student was enthusiastic about specialty and residency
  c. Discuss any inappropriate negative comments about medical school, rotations, specialties, programs, etc.
  d. Discuss if student asked appropriate questions of interviewer about program
4. Professionalism
  a Attire
  b Greeting/handshake/close of interview
