# Supplementary material for: Mock interviews with video-stimulated recall to prepare medical students for residency interviews
Source: MedEdPublish (2016). 2018 Aug 14;7:168. [Version 1] doi: 10.15694/mep.2018.0000168.1 (PMC10701818; doi:10.15694/mep.2018.0000168.1)
Supplement: Peer review history

# Reviewer report: Reviewer response for version 1

**DOI:** 10.21956/mep.19627.r29210

**Published:** 2018-11-23

**Authors:** Ian Wilson

**Affiliations:** 1 International Medical University

This review has been migrated. The reviewer awarded 2 stars out of 5 This is a well written paper on a topic of particular interest to practitioners in the US. It does however have an important message about feedback that is of wider significance.It is let down by a couple of issues. Firstly it relies on participants self perceived changes. There is a large body of literature that indicate that this may not result in change in behaviour.Secondly the instruments used have not been validated in any way. This undermines the power of the study.Finally I have an issue that is outside the ambit of the authors. I have a concern that the ethics committee approved this study. If the intervention is effective this would give an advantage to those who had the intervention when it comes to the selection interview. I would have had a major difficulty in obtaining approval for this study.

Reviewer Expertise:

NA

No decision status is available

# Reviewer report: Reviewer response for version 1

**DOI:** 10.21956/mep.19627.r29212

**Published:** 2018-11-13

**Authors:** Ariela Marshall

**Affiliations:** 1 Mayo Clinic

This review has been migrated. The reviewer awarded 4 stars out of 5 The authors describe a well-designed research study which has the potential to positively impact medical students’ residency interview preparation in the future. The number of students who chose to participate was relatively small and therefore the comparative data from the two groups (video review versus non video review) was limited by statistical power, but there was a demonstrable positive effect of doing the preparatory interview in general. I wonder whether this would have been better designed with a “placebo” arm (i.e. students who choose to participate randomized to either mock interview with video review versus no mock interview at all) to better gauge the overall impact of the mock interview. The fact that all students who chose to participate did receive some interview practice was probably helpful to the students themselves but may dilute the true impact of interview practice versus none when the comparison was limited to one type of feedback versus the other. Nevertheless, this was certainly an interesting study which could be pursued further at the authors’ institution as well as at others. I wish the authors had spent a bit more time on a “next steps” section discussing their plans (or lack thereof) for expansion of the mock interview process to all medical students planning to apply for residency.

Reviewer Expertise:

NA

No decision status is available

# Reviewer report: Reviewer response for version 1

**DOI:** 10.21956/mep.19627.r29209

**Published:** 2018-10-26

**Authors:** Tina Schade Willis

**Affiliations:** 1 Indiana University

This review has been migrated. The reviewer awarded 4 stars out of 5 The authors implemented a feasible process to provide video-stimulated recall interview preparation for residency match. Although small and at a single center, this was nicely done with a straightforward process that could be adapted at other programs. My main criticism is the outcome measure being perception of preparedness rather than some measure of actual performance in residency interviews. This limitation was identified by the authors in the discussion. I am also interested if the program continued to use video-stimulated recall after the study was complete. The time and resources needed for implementing this program would be helpful follow up in for readers that are interested in sustainability of such a mock interview program in medical schools. Overall this was nicely done and reinforces the importance of a standardized approach to residency interview preparation.

Reviewer Expertise:

NA

No decision status is available

# Reviewer report: Reviewer response for version 1

**DOI:** 10.21956/mep.19627.r29211

**Published:** 2018-08-19

**Authors:** Trevor Gibbs

**Affiliations:** 1 AMEE

This review has been migrated. The reviewer awarded 4 stars out of 5 Pre-residency interviews for selection and training have become more common now in many parts of the world, so this paper covers an important area for development. The structure and clarity of the paper is very good and the methodology chosen is appropriate. The conclusion section provided by the authors "says it all" and although I don't feel that the paper introduces us to anything new, it does bring out some very important issues related to the power of effective feedback- a subject or topic that still has more questions than answers. The addition of the video as a method of the faculty showing and clarifying their observations certainly seemed to add to the overall value of the feedback, but I think that the process of feedback, and using faculty, used to structuring effective feedback, made the exercise very worthwhile.I am not sure that the pre and post test questionnaires gave much new information, and as the authors suggest, they gave perceived improvements in confidence; a much more extended evaluation is required in this situation and I hope that the authors can follow this group of students through. I also wondered how much feed- forward was provided to the students; how to improve and move forward.On another note regarding the methodology, I wondered as to the value of collecting so much demographic information- was it really necessary and what extra did it bring to the paper?A good paper for all involved in interviewing to read.

Reviewer Expertise:

NA

No decision status is available
